# How (specific) would you like your T-cells today? Generating T-cell therapeutic function through TCR-gene transfer

**DOI:** 10.3389/fimmu.2012.00186

**Published:** 2012-07-06

**Authors:** Inbal Daniel-Meshulam, Shlomo Ya’akobi, Chen Ankri, Cyrille J. Cohen

**Affiliations:** Laboratory of Tumor Immunology and Immunotherapy, The Mina and Everard Goodman Faculty of Life Sciences, Bar-Ilan University, Ramat Gan, Israel

**Keywords:** T-cell receptor, TCR-gene transfer, T-cells, immunotherapy, cancer, infectious diseases, autoimmunity

## Abstract

T-cells are central players in the immune response against both pathogens and cancer. Their specificity is solely dictated by the T-cell receptor (TCR) they clonally express. As such, the genetic modification of T lymphocytes using pathogen- or cancer-specific TCRs represents an appealing strategy to generate a desired immune response from peripheral blood lymphocytes. Moreover, notable objective clinical responses were observed in terminally ill cancer patients treated with TCR-gene modified cells in several clinical trials conducted recently. Nevertheless, several key aspects of this approach are the object of intensive research aimed at improving the reliability and efficacy of this strategy. Herein, we will survey recent studies in the field of TCR-gene transfer dealing with the improvement of this approach and its application for the treatment of malignant, autoimmune, and infectious diseases.

## INTRODUCTION – TCR-GENE TRANSFER FROM BENCH TO BEDSIDE

As an integral part of the adaptive immune system, T-cells have been attributed several protective functions directed against both microbial pathogens and tumor cells. Although derived from the self, neoplastic cells often express tumor antigens that can discriminate them from normal tissues and can be recognized by the adaptive immune system (Novellino et al., 2005). Indeed, T lymphocytes demonstrate the capacity to eradicate cancer cells and a growing body of studies has shown that the adoptive cell transfer (ACT) of tumor-specific T lymphocytes (Hawkins et al., 2010; Restifo et al., 2012) isolated from the tumor itself has been demonstrated to mediate impressive tumor regression in advanced melanoma patient, with almost a quarter of the treated individuals durable complete responders (Rosenberg et al., 2011). Clinical trials based on adoptive T-cell transfer have been conducted in the last two decades for the treatment of viral conditions (Fujita et al., 2008; Berger et al., 2009), especially in the context of bone marrow transplantation. Initially targeting cytomegalovirus (CMV; Walter et al., 1995), this strategy has been extended to other viruses such as Epstein–Barr virus (EBV; Heslop et al., 1996), adenoviruses (Feuchtinger et al., 2006), and multiple viruses simultaneously (Leen et al., 2006).

Still, the isolation of such antigen-specific T-cells is not always possible and as such, alternative approaches have been designed to enable the generation of antigen-specific lymphocytes from peripheral T-cells. T lymphocytes can recognize their cognate antigen through the binding of their T-cell receptor (TCR) to an epitope presented by major histocompatibility complex (MHC) molecules on the target cells (Vyas et al., 2008). This implies that the TCR dictates the specificity of a given T-cell and that it should prove feasible to provide T-cells with new specificities by transferring the genes of a given TCR. The successful reprogramming of T-cell specificity by TCR-gene transfer was first demonstrated by Steinmetz and colleagues in a murine system (Dembic et al., 1986). Originally, the purpose of this report was to study the receptor dynamics, but it opened a novel field of therapeutic research dealing with gene-mediated redirection of T-cell specificity. This approach was then applied to endow T-cells with tumor specificity using a melanoma-specific TCR *in vitro* (Clay et al., 1999), and subsequently *in vivo*, using an influenza virus-specific receptor (Kessels et al., 2001).

Basically, this strategy relies on the isolation of the genes encoding the α and β chains of a TCR specific for an antigen from a T-cell clone (e.g., from tumor-infiltrating lymphocytes (TILs), peptide-stimulated PBLs from healthy individuals or from immunized human leukocyte antigen (HLA)-transgenic mouse). While the rapid isolation of suitable TCRs for therapy remains a challenge due in part to their high variability (around 40–50 different framework genes for either the α or the β), multiple strategies (mainly using sets of primers for TCR-5′ regions or the rapid-amplification of cDNA ends – RACE) have been published (Aarnoudse et al., 2002; Boria et al., 2008; Birkholz et al., 2009; Walchli et al., 2011). Then, these α and β chains are cloned into an expression vector and transduced to previously stimulated normal peripheral T lymphocytes. This enables the reprogramming of the adaptive immune response against antigens of choice based on the specificity of the introduced TCR (reviewed elsewhere; Schmitt et al., 2009; Udyavar and Geiger, 2010; Thomas et al., 2010). Several clinical studies were elaborated based on this conceptual approach for the treatment of advanced cancer patients: Morgan et al. (2006) demonstrated for the first time that it was possible to transduce normal autologous PBLs from stage IV-metastatic melanoma patients with an MART1-specific TCR and generate large numbers of MART1-specific cells (10^9^–10^10^) to be infused back to the patients. In this Phase I clinical trial, 17 metastatic melanoma patients were treated and 2 of them (12%) demonstrated dramatic tumor regression leading to an objective clinical response. This was followed by a second study in which the same group made use of two high-affinity TCRs against the melanoma antigens MART-1 and gp100 (Johnson et al., 2006) (including an HLA-A2/gp100_154_-specific murine TCR) in a clinical trial (Johnson et al., 2009) in which the objective response (OR) rate raised to 30% of the patients treated. Recently, more clinical studies were aimed at exploring the therapeutic potential of TCR-gene transfer to target other cancers than melanoma, using carcinoembryonic (CEA)- (Parkhurst et al., 2011a), p53- (Davis et al., 2010), and NY-ESO- (Robbins et al., 2011) specific TCRs. The CEA-TCR and p53-TCR were based on murine TCRs isolated in HLA-A2 transgenic hosts and in each study, one patient responded to the treatment. Still, the CEA-TCR clinical trial was limited to only three patients, due to severe inflammatory colitis. Such toxicity emphasizes the possible on-target effects of such therapy. However, Robbins et al. (2011) reported encouraging results using an affinity-enhanced NY-ESO-1-specific TCR. Half (5/11) of the melanoma patients treated as well as 67% of the synovial cell cancer patients underwent an objective clinical response, with two complete responders. Notably, no toxicities were observed in all the patients treated.

Thus far, it has been reported that more than a hundred patients have been treated by the Rosenberg group in the Surgery Branch at NCI using TCR-gene transfer (Park et al., 2011). Nevertheless, several hurdles that may hinder the efficacy of these treatments have been identified along the years and several studies have attempted to solve these issues that include the type of the vector to be used, its configuration, the safety of the procedure, TCR chains mispairing, and the desired functional avidity of the reprogrammed cells. In the present review, we will aim at giving an overview of the recent development in this field and will also elaborate on the development of TCR-gene engineering for conditions other than neoplastic diseases.

## MANIPULATING THE FUNCTIONAL AVIDITY OF THE TCR-ENGINEERED CELLS

**FIGURE 1:**
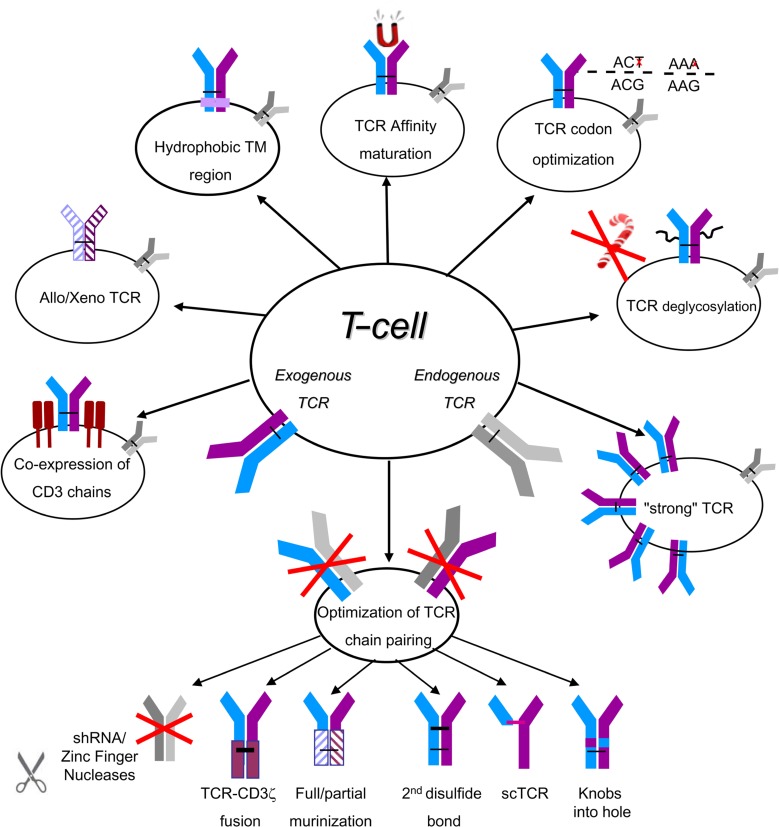
**A summary of optimization strategies for TCR expression and pairing.** The naturally expressed/endogenous TCR is depicted in gray and the introduced/exogenous TCR in blue/purple. TM, transmembrane; sc, single chain.

One of the central questions that pertain to TCR-based gene modifications of lymphocytes is to what extent it is possible for the introduced TCR to reach similar levels of surface expression and functionality as the endogenous one. As T-cell functional avidity is dictated mainly by both TCR affinity and the number of TCR molecules expressed (Schodin et al., 1996), much efforts has been devoted to improving these biophysical properties in TCR-engineered cells using two important approaches: the improvement of TCR affinity and expression and the enhancement of TCR chain pairing and expression. Based on *in vitro* comparative assays (Johnson et al., 2006) and the recent solution of the crystal structure (Borbulevych et al., 2011) of a highly avid (DMF5) and a medium-avid (DMF4) MART1-specific TCRs, and on the other hand the results obtained in two clinical trials published using these TCRs (Morgan et al., 2006; Johnson et al., 2009), it is reasonable to surmise that the use of TCRs that endow T-cells with superior functional avidity might help to improve OR rates [i.e., 30% OR for the DMF5 (Johnson et al., 2009) compared to 12% for the DMF4 (Morgan et al., 2006)]. In addition, TCR affinity increase can assist in augmenting T-cell sensitivity to tumors and in compensating for sub-optimal TCR expression. Such high-affinity TCR should also function in CD8-negative cells such as Th1 or Th17, providing additional support for the anti-tumor response (Cohen et al., 2005; Kuball et al., 2005; Udyavar et al., 2009). Several approaches to increase the functional avidity of TCR-engineered cells have been described lately (summarized in **Figure 1**).

### TCR AFFINITY MATURATION

Because most of the tumor antigens are self antigens, the isolation of high-affinity TCR reactive against tumor antigen from human donors can represent a major challenge, since high-avidity CTLs specific for tumor cells may be deleted by negative selection. However, it is possible to increase TCR affinity by mutating selectively amino acids in polymorphic TCR complementarily determining regions – CDRs (Chlewicki et al., 2005). The screening of mutated TCRs using yeast or phage-display libraries can yield affinity improvement up to supra-physiological levels (Holler et al., 2000; Li et al., 2005). For example, Li et al. (2005) isolated NY-ESO-specific TCR with affinities in the picomolar range. Similarly, a Gag-specific TCR that underwent a 360-fold increase in affinity demonstrated a more efficient control of the spread of HIV virus *in vitro *(Varela-Rohena et al., 2008). Nevertheless, such affinity increase can prove detrimental to T-cell function by endowing T-cells with non-specific/self-reactivities (Zhao et al., 2007a) or, paradoxically, by causing defective recognition of low concentrations of antigen (Thomas et al., 2011). In parallel, some studies demonstrated the benefit of introducing a reduced number of CDR-targeted mutations; Robbins et al. (2008) increased the affinity of several TCR by several fold using an alanine-scan of CDR regions and site-directed mutagenesis of only one to two residues. This approach was used to increase the affinity of two TCRs, a CEA- and an NY-ESO1-specific TCRs, which were subsequently used in recent clinical trials (Parkhurst et al., 2011b; Robbins et al., 2011) in which several patients underwent clinical objective tumor regression. Another work recently showed that TCRs seldom express a glycine residue at position 107 in the CDR3β instead of serine. Based on modeling and molecular dynamics simulation, a G107S replacement led to 10–1,000-fold increase in antigen sensitivity in three of four TCRs tested (Alli et al., 2011).

### ISOLATION OF TCRs FROM HLA-MISMATCHED- OR TRANSGENIC DONORS

High-affinity TCRs could be isolated from HLA-mismatched donors (Sadovnikova and Stauss, 1996; Savage et al., 2004; Amir et al., 2011), HLA-transgenic mice (Theobald et al., 1995) or transgenic mice expressing the human TCR repertoire (Li et al., 2010), as logically, tolerance mechanisms should not influence non-self reactivity, provided the targeted MHC/peptide complex is recognized as foreign. Additionally, several models utilized HLA-A2 transgenic mice to isolate TCRs, which were revealed to display CD8-independent characteristics (Sherman et al., 1992). We and others showed that such murine TCRs can efficiently function in CD4^+^ cells (Cohen et al., 2005; Kuball et al., 2005; Johnson et al., 2009).

### TCR CODON OPTIMIZATION

The genetic code bears some degrees of degeneration as a defined amino acid can be encoded by several (synonymous) codons, which are differentially represented in the cell. Moreover, cryptic splicing sites, mRNA secondary structure, and instability motifs can reduce the expression of proteins. Using algorithms to modify the DNA sequence of TCRs “silently,” several TCRs have been “codon-optimized” leading to higher expression levels and enhanced reactivity both *in vitro* and *in vivo* (Scholten et al., 2006; Jorritsma et al., 2007).

### TCR DEGLYCOSYLATION

Based on the fact that TCR glycosylation can reduce TCR expression and favor its internalization (Daniels et al., 2002), Kuball et al. (2009) demonstrated that the deletion of some of these N-glycosylation sites (4–5 total in the constant domain) from either human or murine TCRs increased the functional avidity of T-cells transduced with these mutated TCRs.

### USE OF “STRONG” TCRs

It appears that certain TCRs (termed “strong TCRs”) can compete better for surface expression when expressed in the presence of various other TCRs (Sommermeyer et al., 2006; Heemskerk et al., 2007). TCR stability is likely to be influenced by protein dynamics and folding as well as interactions between the TCR-variable regions. However, it is unclear what determines the “strength” of a defined TCR *a priori*.

### INCORPORATION OF HYDROPHOBIC MUTATIONS IN THE TRANSMEMBRANE REGION

The TCR transmembrane regions contain three positively charged residues that may be associated with the lack of stability of the entire chains (Soetandyo et al., 2010). We designed an original approach to selectively improve exogenous TCR stability by increasing the hydrophobic nature of the TCRα transmembrane region. Incorporation of hydrophobic residues at evolutionary-permissive positions resulted in an enhanced surface expression of the TCR chains, leading to an improved cellular avidity and anti-tumor TCR activity (Haga-Friedman et al., 2012).

### OPTIMIZATION OF TCR CHAINS PAIRING

One of the central impediments in the TCR-gene transfer strategy lies with the mispairing of the exogenous α and β TCR chains (α_EX_ and β_EX_) with the naturally expressed endogenous TCR chains (α_NAT_ and β_NAT_; reviewed in detail in Govers et al., 2010). Thus, four different αβ dimers can form in the transduced cells: the endogenous TCR (α_NAT_/β_NAT_), the transduced (desired) TCR (α_EX_/β_EX_) as well as two mixed dimers (α_EX_/β_NAT_ and α_NAT_/β_EX_). Since the surface expression of these TCR necessitates for these chains to assembly with a limited number of CD3 molecules, the existence of unproductive forms of TCR leads to reduced levels of the exogenous TCR. Additionally, two reports recently showed that these TCRs mixed heterodimers may engender autoimmunity manifestations and self-reactivity both in a mouse model (Bendle et al., 2010) and *in vitro* (van Loenen et al., 2010).

Consequently, many groups, including ours, are involved in developing approaches to reduce the mispairing effect as well as to promote the pairing of the exogenous TCR chains. These strategies include the addition of a second disulfide bond (Cohen et al., 2007; Kuball et al., 2007), the “murinization” of all or part of the TCR constant regions (Stanislawski et al., 2001; Cohen et al., 2006; Voss et al., 2006; Thomas et al., 2007; Bialer et al., 2010; Sommermeyer and Uckert, 2010), the use of a “knob into holes” approach (Voss et al., 2008), of chimeric TCR-CD3ζ chain (Sebestyen et al., 2008; Govers et al., 2011) or of single-chain TCRs (Chung et al., 1994; Voss et al., 2010; Aggen et al., 2012) and have been described in details in several reviews (Govers et al., 2010; Thomas et al., 2010; Merhavi-Shoham et al., 2012). In addition, shRNA sequences can be incorporated into the TCR encoding vector to knock down the expression of the endogenous TCR (Okamoto et al., 2009). Lately, another elegant approach to knock down the endogenous TCR was reported and is based on the use of zinc-finger nucleases (ZFNs) that target the endogenous TCRα and β chains (Provasi et al., 2012).

### CO-EXPRESSION OF CD3 CHAINS

The restricted level of CD3 molecules can represent a bottleneck for TCR expression. Ahmadi et al. (2011) demonstrated that increasing the expression of CD3 chains (using polycistronic vectors) can boost the functional avidity of TCR-transduced T-cells and their *in vivo* performance.

## GENETIC APPROACHES AND TOOLS FOR THE ENGINEERING OF T-CELLS

The genetic modification of T-cells with a TCR necessitates reliable platforms for the transfer of the genetic information. To date, most of the platforms used for TCR-gene transfer, both for research and in clinical settings, were based on γ-retroviral vectors such as the mouse stem cell virus (MSCV) or myeloproliferative sarcoma virus (MPSV; Morgan et al., 2006; Uckert and Schumacher, 2009). γ-retroviral vectors mediate genome integration of the transgene(s), which enables its long-term expression in the transduced cells. In this type of vectors, the exact cloning position of the transgene relative to *cis*-elements in the retroviral construct appears critical for proper expression of the TCR (Frankel et al., 2011). Also, retroviral vectors may demonstrate a risk of insertional mutagenesis, which can cause dysregulated gene expression and subsequent malignant transformation (Hacein-Bey-Abina et al., 2003). However, it is important to note that all the recent TCR-gene transfer clinical trials were based on the use of MSCV-based retroviral vectors (Park et al., 2011) and that no signs of lymphoproliferative disease were noted in the patients treated. Additionally, a comprehensive body of evidence support the notion that no replicative competent retroviruses (RCR) or adverse events have been observed in a considerable number of clinical trials (Bear et al., 2012). Still, primary lymphocyte stimulation is essential when using γ-retroviral vectors and the latter may shift the T-cell phenotype causing some levels of T-cells exhaustion (Gattinoni et al., 2005; Hinrichs et al., 2011). Interestingly, the TCR transgene expression in patients seems to decrease quite rapidly (1–2 weeks) following adoptive transfer unlike what was observed in *in vitro* conditions, where it can last several weeks (Burns et al., 2009). This decrease seems to be associated with lower general transcriptional activity of the transduced T-cells, in a similar way as for the endogenous TCR (van Loenen et al., 2011). However, this situation may be reversed when restimulating transduced cells.

It is also possible to utilize lentiviral platforms (Tsuji et al., 2005; Yang et al., 2008; Circosta et al., 2009; Jones et al., 2009) as they may provide the benefit of efficiently transducing non-dividing cells with a safer integration profile (Levine et al., 2006) and resistance to silencing (Frecha et al., 2010). Nonetheless, the use of stimulating factors such as cytokines is required to achieve desirable levels of TCR expression (Cavalieri et al., 2003; Jones et al., 2009; Perro et al., 2010). In mouse settings, a recent comparison of lenti- and retroviral vectors showed that the latter mediated better results in TCR-gene transfer assays (Kerkar et al., 2011).

Non-viral means to engineer T-cells would significantly reduce the need for intensive testing of the viral supernatant, reducing production time. As such, the use of *in vitro*-transcribed RNA molecules introduced by electroporation (Schaft et al., 2006a; Zhao et al., 2006a) or DNA constructs (Till et al., 2008) represents an interesting alternative for the gene modification of lymphocytes, not only for the rapid screening of gene function (Bialer et al., 2010; Almasbak et al., 2011) but also in potentially therapeutic settings (Zhao et al., 2010). On the other hand, the rapid disappearance of transgene after a few days (Zhao et al., 2006a) inherent to this transient system represents a major disadvantage of this approach. Another interesting non-viral approach is based on the use of the *Sleeping Beauty* transposon system (Ivics et al., 1997; Huang et al., 2006). In this system that was “resurrected” based on fossil sequences found in the genomes of salmonid fish, the gene(s) of interest are integrated into the genome using a transposase. This system necessitates the concomitant transfer of two nucleic acid polymers, one being the transposon encoding the gene(s) of interest and the other the transposase itself. In the context of TCR-gene transfer, the use of this approach has recently been reported to provide human T-cells with tumor specificity (Peng et al., 2009; Jin et al., 2011).

Beyond the type of vector to be used, it is important to note that as the TCR is a dimer formed of two chains, TCRα and TCRβ, these have to be expressed simultaneously by the engineered cells. As the separate introduction of both chains in the recipient chains has proven difficult and rather ineffective, several vector designs strategies have been elaborated to facilitate the efficient expression of the TCR dimer (reviewed in Uckert and Schumacher, 2009). The two main strategies that have been utilized clinically rely on the use of either an internal ribosomal entry site (IRES) or a 2A peptide sequence separating the two chains. One of the potential drawbacks related to the use of an IRES sequence is the possibility that the gene downstream of the IRES is expressed at a lesser level (Ghattas et al., 1991; Mizuguchi et al., 2000). Alternatively, a 2A peptide sequence, which is employed by several viruses such as the picornavirus (Szymczak and Vignali, 2005), provide an effective way to express simultaneously multiple proteins. Indeed, it has been showed that the 2A peptide can promote a ribosomal “skip.” We also showed that a furin cleavage site as well as an amino-acid spacer can be added to the 2A sequence to enhance transgene expression (Yang et al., 2008). When comparing both approaches (IRES or 2A peptide) it has been showed that the latter may be better for efficient TCR expression (Leisegang et al., 2008).

Though most of the TCR-gene transfer studies are based on αβTCRs targeting an peptide/MHC class I complex, some reports have also made used of other types of receptors to target tumor cells both *in vitro* and* in vivo*: TCR specific for minor antigens (Heemskerk et al., 2003; Mommaas et al., 2005), MHC II-restricted TCRs (Yang and Baltimore, 2005; Zhao et al., 2006b; Ha et al., 2010; Kerkar et al., 2011; Straetemans et al., 2012), and γδTCR (Marcu-Malina et al., 2011).

TCRs is generally thought to be restricted to a single epitope (not considering altered peptide ligands). Therefore, the possibility that a single TCR would target multiple cancer epitopes simultaneously would be advantageous. In that regard, an αβTCR targeting originally an HLA-A0201-restricted MAGE-A3 epitope was recently isolated and interestingly, it demonstrated reactivity against multiple MAGE epitopes (Chinnasamy et al., 2011).

Additionally and beyond our present scope, it is also possible to modify T-cells to express genes other than those encoding a TCR to redirect T-cell function. These include antibody-based chimeric-antigen receptor (CAR) and other genes to modulate the quality of transduced T-cells (reviewed in Kershaw et al., 2005; Jena et al., 2010; Kohn et al., 2011; Merhavi-Shoham et al., 2012). Another approach for the direction of T-cell specificity was reported lately and is based on bi-specific molecules termed immune-mobilizing monoclonal TCRs against cancer (ImmTACs; Liddy et al., 2012). These newly developed reagents comprise an scFv specific for CD3 (T-cell binding moiety) and a high-affinity cancer-specific TCR (tumor targeting moiety) and were shown to mediate tumor recognition and killing both *in vitro *and *in vivo* (Liddy et al., 2012).

## WHICH CELL TO ENGINEER?

One of the basic assumptions when it comes to TCR engineering for cancer treatment is that since malignant cells ought to present their tumor antigens via the MHC class I molecules, the appropriate effector cells to be genetically modified ought to be CD8^+^ T lymphocytes. Nonetheless, due to the complexity of the acquired immune system and the different T-cell functional subsets, recent work has been devoted to the importance of the type/subset of T-cells to be adoptively transferred.

While they represent a minority of the circulating lymphocytes, γδ T-cells have the advantage of expressing TCR chains that apparently do not pair with αβTCR chains (Saito et al., 1988). Thus, the use of these cells represents an interesting possibility to prevent the mispairing of the naturally expressed TCR chains with the exogenous one (van der Veken et al., 2006). Bi-specific T-cells represent another possible option (reviewed in Marr et al., 2012); for example, virus-specific cells (specific for EBV, CMV or Influenza) can be engineered to express an additional receptor to target tumor cells (Rossig et al., 2002; Murphy et al., 2007; Pule et al., 2008; van der Veken et al., 2009). The use of these cells may considerably reduce off-target effects as these cells have a defined specificity and can provide protection from latent viruses during the immunosuppressed phase prior to adoptive transfer. In addition, the continuous expression of viral antigens following initial infection (such as in the case of EBV) may provide constant stimulation leading to increased persistence (Pule et al., 2008).

We and others have also attempted at modifying precursor cells such as hematopoietic stem cells (HSCs; van Lent et al., 2007; Zhao et al., 2007b), or more recently induced pluripotent stem cells (iPS; Lei et al., 2011), to express a TCR and to be further differentiated into T-cells using, for example, the OP9-DL1 system. Their plasticity, differentiation capability into many T-cell subsets, and the possible increased potential to persist in the host (correlated with clinical response; Robbins et al., 2004; Morgan et al., 2006) represent a formidable advantage over peripheral T-cells and it was shown that such modified cells demonstrate anti-tumor function *in vivo* (Alajez et al., 2005; Yang and Baltimore, 2005; Ha et al., 2010). However, one has to bear in mind that a major drawback lies with their possible transformation induced by retroviral vectors (Hacein-Bey-Abina et al., 2003), though the latter might be circumvented using lentiviral- or transposon-based platforms.

Another interesting option is the use of certain subsets of differentiated T-cells. Following activation, naïve T-cells can turn into effector cells, effector memory or central memory cells. T-cell populations derived from the latter were shown to be exquisitely persistent over time, to further differentiate into both effector and central memory *in vivo*, with adequate response to antigenic challenge, both in a macaque model (Berger et al., 2008) and more recently for human virus-specific cells in immunodeficient mice (Wang et al., 2011). In regard to TCR-transduced cells, these cells generated *ex vivo* exhibit mainly a CD62L^+^/CD45RO^+^ phenotype similar to central memory T-cells (Yang et al., 2011). Still, Hinrichs et al. (2009),^2011^ showed that TCR-transduced naïve T lymphocytes display increased transgene expression and proliferation compared to memory cells. Muranski et al. (2011) also found that Th17-polarized CD4+ T-cells demonstrate enhanced anti-tumor function, resistance to apoptosis and persistence following adoptive transfer with stem-cell like multipotency signature. The role of Th17 cells in mediating tumor regression was also recently highlighted in a study dealing with the identification of tumor antigens able to mediate tumor regression following immunization (Pulido et al., 2012). Interestingly, Gattinoni et al. (2011) recently identified a subset of T-cells (CD45RO^–^, CCR7^+^, CD45RA^+^, CD62L^+^, CD27^+^, CD28^+^, and IL-7Rα^+^) that are endowed with both naïve and memory properties which they termed T memory stem cells (T_scm_). This fascinating subset seems more potent in ACT settings than naïve, central, or effector memory T lymphocytes. Despite their rarity, this type of cells can be generated *in vitro* by stimulating naïve T-cells in the presence of TWS119, a Wnt pathway activator (Gattinoni et al., 2009,^2011^) and based on their potential, these cells might provide a promising subset to be TCR gene engineered, which would demonstrate increased reactivity and persistence over time in patients.

## SAFETY ISSUES

Notwithstanding classical gene therapy-related issues that some of them will be discussed below, manipulating the immune system and often bypassing tolerance mechanisms can generate immune toxicities. Previous works have shown that the transfer of tumor antigen-specific T-cells can cause autoimmune manifestations such as ocular toxicity (Palmer et al., 2008) or systemic autoimmunity (Rosenberg et al., 1988) and this subject has been thoroughly reviewed in Amos et al. (2011).

Still, one the obvious advantages of the use of TCR-engineered cells in the published clinical trials and pre-clinical studies is their autologous origin, which certainly eases their engraftment and long-term persistence. Nevertheless, the expression of transgenes mainly from xenogenic provenance (e.g., murine TCRs or virus-derived 2A peptides) might trigger immunogenicity. Davis et al. (2010) showed that a little less than a quarter of the patients administered T-cells expressing fully murine TCRs (specific for a human pMHC complex) indeed developed antibodies directed essentially to the TCR-variable regions. However this response was not associated with the level of transduced cell persistence or response to therapy.

A comprehensive study showed that 9 years following gene modification of T-cells with a retrovirally expressed transgene, these cells demonstrated stable gene expression profiles and phenotype with no evidence of clonal selection (Recchia et al., 2006). Additionally, it seems so far that no off-target or GVH disorders were observed in patients treated at the Surgery branch (NCI) in seven different TCR-gene transfer clinical protocols (three of which made use of murine TCR that preferentially pair; Park et al., 2011). As quoted above, no replication competent viruses occurrence was observed in hundreds of patients treated with retrovirally engineered T-cells (Bear et al., 2012).

Nevertheless, one has to bear in mind the possible risks associated with this kind of gene therapy such as reactivity to normal tissues expressing the targeted antigen (Johnson et al., 2009; Parkhurst et al., 2011a), possible newly generated specificities associated with TCR mispairing (Bendle et al., 2010; van Loenen et al., 2010), and insertional mutagenesis. Several approaches have been developed to readily eliminate engineered T-cells in case such adverse events take place. These include in part the inclusion of suicide genes (Morgan, 2012) such as a inducible Caspase 9 molecular switch (Straathof et al., 2005; de Witte et al., 2008; Di Stasi et al., 2011) or Herpes Simplex Virus thymidine kinase (HSV-TK; Bonini et al., 1997) in the vector and subsequent triggering of cellular death using a chemical inducer of dimerization (CID) or Ganciclovir, respectively. It is also possible to deplete *in vivo* TCR-expressing T-cells using antibodies directed against specific tags (such as c-myc or HA), provided the cDNA sequences encoding these peptide tags are added to the 5′-end of the gene(s) encoding the TCR chains (Kieback et al., 2008). In addition, this particular TCR-tagging strategy saves the need to include an additional (suicide) gene in the vector construct.

## BEYOND CANCER: TCR-GENE TRANSFER FOR OTHER DISORDERS

The immune system in general, and T-cells in particular, have been shown to be critically involved both in host defense and unfortunately, in immunopathologies (Morris and Allen, 2012). Thus, the genetic modification of T lymphocytes can provide an attractive way to alter or redirect the immune response in order to target pathogens or remedy to certain T cell-linked pathologies (**Table 1**).

**TABLE 1:** Use of TCR-gene transfer for non-neoplastic diseases.

Condition	Disorder/Pathogen	References
Autoimmunity	Arthritis	Fujio et al. (2006)
		Wright et al. (2009)
	Systemic autoimmune disease	Fujio et al. (2004)
Viral infection	Cytomegalovirus (CMV)	Schub et al. (2009)
	Epstein-Barr virus (EBV)	Schaft et al. (2006b)
		Hart et al. (2008)
	Hepatitis B virus (HBV)	Gehring et al. (2011)
	Hepatitis C virus (HCV)	Zhang et al. (2010)
	Human immunodeficiency	Cooper et al. (2000)
	virus (HIV)	Ueno et al. (2004)
		Varela-Rohena et al. (2008)
	Human papilloma virus (HPV)	Scholten et al. (2005, 2011)
	Influenza virus	Kessels et al. (2001)
Microbial	*Mycobacterium*	Luo et al. (2011)
infection	*tuberculosis*	

Amongst T-cells, the Treg subset is important for the immunomodulation of self-reactivity, especially in the case of autoimmune disorders. It is possible to generate antigen-specific Tregs by gene transfer and defined T-cells with regulatory activity, in order to treat autoimmune disease. Several groups showed that co-transduction of T-cells with FOXP3 and specific TCR created T regulatory cells that were able to suppress arthritis in different models (Fujio et al., 2006; Wright et al., 2009), for example, by reduction of Th17 cells and decrease of bone destruction (Wright et al., 2009). Still in this study, TCR-redirected natural Tregs showed a better therapeutic potential than FOXP3-transduced cells. In addition, redirected regulatory T-cells specific for nucleosome antigen were shown to suppress a systemic autoimmune disease in a mouse model (Fujio et al., 2004).

TCR-gene transfer approaches could also be implemented for the redirection of T-cell response against HIV. For example, Cooper et al. (2000) showed more than a decade ago that T-cells can be transduced with a GAG-specific TCR and target cells displaying the relevant epitope. Other HIV antigens were also targeted similarly by TCR-transduced T-cells specific for POL. The engineered lymphocytes showed potent inhibitory activity against HIV-1 replication *in vitro* and substantial cytotoxic activity and cytokine production triggered by either epitope-pulsed or infected with HIV-1 target cells (the functional phenotype was similar to those of the parental CTL clone) (Ueno et al., 2004). Using an affinity-enhanced codon-optimized GAG-specific TCR, Varela-Rohena et al. (2008) were able to demonstrate very high level of TCR expression that translated into a high sensitivity to the presence of low levels of cognate epitopes on APCs, including escape mutants.

Additionally, recent studies have focused on the possibility of engineering T-cells to recognize other viral epitopes. For example, a CD8-independent HCV-specific TCR was isolated and conferred reactivity to both CD4 and CD8 T-cells, leading to the recognition of HCV^+^ hepatoma cells (Zhang et al., 2010). Other reports have exemplified the potential of TCR-gene transfer strategy to target CMV (Schub et al., 2009), EBV (Schaft et al., 2006b; Hart et al., 2008), HBV (Gehring et al., 2011), and HPV (Scholten et al., 2005,^2011^).

Lately, this approach has been extended to bacterial antigens. As adoptive transfer of *Mycobacterium tuberculosis*-specific effector T-cells has been shown to confer immunity to infected recipients (Woodworth et al., 2008; Duffy et al., 2009), Luo et al. (2011) aimed at adapting TCR-gene transfer approach to generate anti-bacterial immunity. They isolated *M. tuberculosis* 38-kDa antigen specific HLA class I and class II-restricted TCRs and subsequently minimally murinized their constant regions (Bialer et al., 2010; Sommermeyer and Uckert, 2010) to enhance their expression and function. When introduced in CD8^+^ and CD4^+^ T-cells, respectively, these TCRs where shown to trigger specific cytokine secretion by T-cells in co-culture with antigen-pulsed DCs, as well as cell-mediated cytotoxicity (by the CD8^+^ population) (Luo et al., 2011).

## CONCLUSIONS AND FUTURE DIRECTIONS

TCR-gene modifications of primary human T-cells have demonstrated over the past decade significant achievements, the most important one being its encouraging translation from bench to bedside. In the past few years, successful gene-therapy studies have renewed hopes with regard to the ability to genetically modify patient cells for therapeutic purposes (Kay, 2011; Sheridan, 2011). Unlike for monogenic diseases, the broader implementation of TCR-gene transfer as a semi-personalized medicine approach would require the isolation and the characterization of multiple TCRs, due to HLA polymorphism and the variety of tumor antigens that would need to be targeted. The nature of the antigen, its exquisite expression by the tumor, but more importantly, its immunogenicity and its capacity to evoke efficient tumor response will be a crucial determinant for the success of this kind of therapy. More needs to be understood about antigen immunodominance and the kinetics and dynamics of tumor antigen expression, even if impressive tumor regressions were observed in patients treated with monospecific engineered T-cells (Morgan et al., 2006; Johnson et al., 2009; Park et al., 2011; Robbins et al., 2011). In this regard, extensive screening of tumor reactive T-cells such as TILs may provide valuable information about the specificity of the anti-tumor T-cell response in cancer patients (Andersen et al., 2012). Additionally, the functional characterization of the ability of antigens to mediate tumor regression will provide critical insight as to which epitope(s) TCRs should be specific for (Pulido et al., 2012). Needless to say that the widespread implementation of such “off-the shelf” gene-therapy protocols will benefit from the development of approaches for *in vivo* infection as well as from a better understanding of the cell selection and conditioning process. The combination of newly immunomodulating agents and/or vaccination strategy may improve the clinical response rates (Mellman et al., 2011). Overall, the ability of redirecting the specificity of T-cells genetically holds great promise for the immunotherapy of cancer and other diseases.

## Conflict of Interest Statement

The authors declare that the research was conducted in the absence of any commercial or financial relationships that could be construed as a potential conflict of interest.

## Abbreviations

CTL: cytotoxic T-cells
HLA: human leukocyte antigen
MHC: major histocompatibility complex
OR: objective response
TCR: T-cell receptor
